# A brief intervention to improve exercising in patients with schizophrenia: a controlled pilot study with mental contrasting and implementation intentions (MCII)

**DOI:** 10.1186/s12888-015-0513-y

**Published:** 2015-09-03

**Authors:** Pascal Sailer, Frank Wieber, Karl Pröpster, Steffen Stoewer, Daniel Nischk, Franz Volk, Michael Odenwald

**Affiliations:** Department of Psychology, University of Konstanz, Universitätsstrasse 10, 78464 Konstanz, Germany; Center for Psychiatry, Feursteinstrasse 55, 78479 Reichenau, Germany; Thurgau Psychiatric Services, Seeblickstrasse 3, 8596 Münsterlingen, Switzerland

**Keywords:** Exercise, Implementation intentions, Mental contrasting, Physical activity, Schizophrenia, Self-regulation

## Abstract

**Background:**

Regular exercise can have positive effects on both the physical and mental health of individuals with schizophrenia. However, deficits in cognition, perception, affect, and volition make it especially difficult for people with schizophrenia to plan and follow through with their exercising intentions, as indicated by poor attendance and high drop-out rates in prior studies. Mental Contrasting and Implementation Intentions (MCII) is a well-established strategy to support the enactment of intended actions. This pilot study tests whether MCII helps people with schizophrenia in highly structured or autonomy-focused clinical hospital settings to translate their exercising intentions into action.

**Methods:**

Thirty-six inpatients (eleven women) with a mean age of 30.89 years (*SD* = 11.41) diagnosed with schizophrenia spectrum disorders from specialized highly structured or autonomy-focused wards were randomly assigned to two intervention groups. In the equal contact goal intention control condition, patients read an informative text about physical activity; they then set and wrote down the goal to attend jogging sessions. In the MCII experimental condition, patients read the same informative text and then worked through the MCII strategy. We hypothesized that MCII would increase attendance and persistence relative to the control condition over the course of four weeks and this will be especially be the case when applied in an autonomy-focused setting compared to when applied in a highly structured setting.

**Results:**

When applied in autonomy-focused settings, MCII increased attendance and persistence in jogging group sessions relative to the control condition. In the highly structured setting, no differences between conditions were found, most likely due to a ceiling effect. These results remained even when adjusting for group differences in the pre-intervention scores for the control variables depression (BDI), physical activity (IPAQ), weight (BMI), age, and education. Whereas commitment and physical activity apart from the jogging sessions remained stable over the course of the treatment, depression and negative symptoms were reduced. There were no differences in pre-post treatment changes between intervention groups.

**Conclusions:**

The intervention in the present study provides initial support for the hypothesis that MCII helps patients to translate their exercising intentions into real-life behavior even in autonomously-focused settings without social control.

**Trial registration:**

ClinicalTrials.gov ID; URL: NCT01547026 Registered 3 March 2012.

**Electronic supplementary material:**

The online version of this article (doi:10.1186/s12888-015-0513-y) contains supplementary material, which is available to authorized users.

## Background

Schizophrenia is one of the most debilitating psychiatric disorders; it is frequently linked to long-term disability and a high burden on individuals, families, and societies [63]. The common, persistent, and disabling negative symptoms of schizophrenia include affective flattening, alogia, and avolition [3]. As a result of these symptoms and other barriers (such as cardio-metabolic morbidity and the side-effects of antipsychotic drugs), individuals with schizophrenia spend significantly more time sleeping and sedentary than the general population [28, 48, 60, 61]. Schizophrenia-related metabolic factors and the weight gain produced by antipsychotic drugs are also important contributors to the higher risk of medical illness in people with schizophrenia [13].

Exercising has been found to be an effective way to combat these health risks, with a positive effect on the physical and mental health of individuals with schizophrenia (reviews by [46, 49, 25, 57]; see also [16]). However, a positive attitude towards physical activity and the intention to be more physically active [19] are not sufficient to ensure actual exercising, as indicated by low attendance rates (e.g., 43-91 % attendance rates; [10, 18, 38]) and high dropout rates (e.g., 26 % after three weeks; [18]) in prior physical activity intervention programs. These numbers exemplify the two problems of getting started and staying on track that have been found to be central to successful goal striving [24, 21–23]. Interventions have often focused on the removal of external obstacles such as the lack of free access to fitness facilities [6] or unavoidable reasons for missing a session [58], but the intention-behavior gap between the intentions of patients with schizophrenia to exercise and their actual behavior has remained. In fact, this intention-behavior gap has been found to be pervasive and to occur in a wide range of domains and populations (e.g., [24, 54]).

In light of the importance of physical activity and the pervasiveness and tenacity of the gap between the intention to exercise and actual behavior, the present research examined whether the brief CBT-based self-regulation intervention technique Mental Contrasting and Implementation Intentions (MCII; [41, 44]) can help patients with schizophrenia to translate their intention to exercise into actual exercising behavior. We examined participants’ attendance at regularly offered jogging sessions in clinical settings in which patients’ actions are regulated by highly structured treatment programs and in settings in which individuals can and must autonomously choose their actions.

## Mental contrasting and implementation intentions (MCII)

The theory-based Mental Contrasting and Implementation Intentions approach has been found to be an effective and easily applicable self-regulation strategy to address typical problems of goal striving, such as getting started with an intended behavior or staying on track despite obstacles (overviews by [40, 41, 44]).

With MCII, participants first identify a personal wish or goal (e.g., being physically fit), identify and imagine the most positive future outcomes of goal attainment (e.g., being healthier), and mentally contrast the most positive future outcome with the primary personal obstacle currently impeding their goal attainment (e.g., feeling too tired to exercise). Next, they search for instrumental means to overcome the obstacle and form implementation intentions specifying when, where, and how they want to strive for their personal goal in an if-then format (e.g., “If I feel too tired to exercise, then I will tell myself ‘You can do it!’ and go for a quick run.”).

The MCII strategy has been found to support goal attainment through non-conscious cognitive and motivational processes. The approach increases the accessibility of the mental representations of the critical situations as well as the strength of the implicit associations between the personal goal, the obstacle, and the instrumental means to overcome the obstacle [1, 2, 31, 30, 45, 65–67]. Moreover, it increases implicit (systolic blood pressure) and explicit (self-reported) energization, when the chances of successfully realizing the future outcome are expected to be high (e.g., [42, 53]). These non-conscious processes should allow MCII to support goal attainment even in populations that are known to experience great difficulties in acting on their intentions and whose cognitive functioning is decreased. In line with this assumption, MCII helped chronic back pain patients to increase their physical performance in standardized lifting and ergometer tests 3 weeks and 3 months after discharge above and beyond the usual treatment [14]. Building on these findings, we predicted that MCII could be effectively applied by therapists to help patients with schizophrenia spectrum disorders to become more physically active.

### Social context effects: highly structured vs. Autonomy-focused settings

A patient’s insight into his or her disorder and its treatment is an important prognostic factor for the positive course of schizophrenia [52]. To promote such insight, treatment approaches should allow patients to take responsibility for themselves rather than being highly regulated and therapist-controlled [26, 29]. Living in non-institutionalized settings is one way to promote patients’ responsibility. However, it also creates higher demands on individuals’ self-organization and self-regulation. Given that MCII supports individuals’ self-regulation, we assumed as secondary outcome that the strategy’s effects on physical activity (i.e., attending a jogging program) in people with schizophrenia should be particularly pronounced in settings in which people can and must autonomously choose their actions independent of norms relative to settings in which people are regulated by highly structured treatment programs that prescribe and reinforce normative actions [39, 43]. This additional prediction could be validated by evidence of a Group (MCII vs. control) x Setting (autonomy-focused vs. highly structured treatment program) interaction effect.

## Method

### Design, settings, and participants

#### Design

This multi-center pilot intervention trial studied participation in a jogging program for patients with schizophrenia. In our 2 × 2 between-subjects design, patients were recruited from two types of wards with different degrees of autonomy (quasi-experimental variation) and allocated to two conditions (intervention vs. control; experimental variation).

#### Settings

The study involved three specialized wards in two psychiatric hospitals (Reichenau, Germany and Münsterlingen, Switzerland) that offer inpatient and outpatient treatment to patients with schizophrenia spectrum disorders but do not treat patients with substance-induced psychotic disorders. In these three open and non-acute wards, patients recovering from a recent psychotic episode receive standard treatment according to current guidelines, including pharmacotherapy, individual and group psychotherapy, and occupational therapy, among other treatment regimes. In general, patients are admitted to these wards after several weeks of acute treatment once they have regained stability and are non-suicidal; patients in the early phases of psychotic episodes can also be directly admitted. Ward 1 (Reichenau) has a highly structured environment involving intense therapeutic efforts to activate patients and 24/7 availability of psychiatric care. Wards 2 and 3 (Reichenau and Münsterlingen) focus more on autonomy and self-supply, with daytime care from a team of nurses, medical doctors, and psychologists. These latter wards are intended for early interventions and mainly treat young patients, whereas Ward 1 has no age-based specialization.

#### Participants

The sample of the present study consisted of 36 inpatients (11 female) with a mean age of 30.89 years (*SD* = 11.41) and a diagnosis of a schizophrenia spectrum disorder (Ward 1: *n* = 20; Ward 2: *n* = 10; Ward 3: *n* = 6). Of these 36 participants, twelve had completed post-secondary education, seven had completed secondary education, fifteen had completed compulsory education, and two had not graduated. On average, participants had spent 11.78 years (*SD* = 3.15) in school. The baseline psychiatric characteristics of participants are presented in Table 1. Fourteen percent of the participants had an acute or transient psychotic disorder, showing the same limitations in cognition, perception, affect, and volition as patients with chronic forms of schizophrenia. This percentage did not differ between the studied groups. Participants’ baseline physiological characteristics are described in the results section.

**Table 1:** Socio-demographic and psychiatric characteristics at baseline

		All participants *N* = 36 *N* or *M* (*SD*)	MCII group *N* = 19 *N* or *M* (*SD*)	Control group *N* = 17 *N* or *M* (*SD*)	*p*
Gender	male	25	12	13	.39
	female	11	7	4	
Main Diagnosis					
Paranoid Schizophrenia		20	12	8	
Persistent Delusional Disorders		1	1	0	
Acute and Transient Psychotic Disorders		5	2	3	
Schizoaffective Disorders		10	4	6	
First Episode		11	7	4	.39
PANSS Negative Score		19.1 (6.0)	18.1 (5.7)	20.2 (6.3)	.31
BDI-II		13.8 (8.5)	16.1 (9.4)	11.2 (6.9)	.09
Prescribed Medications					
Chlorpromazine Equivalent x100mg		7.0 (4.9)	6.3 (3.5)	7.9 (6.1)	.32
SSRIs		3	2	1	
Benzodiazepines		7	4	3	
Mood Stabilizers		3	1	2	
Anticholinergics		7	6	1	

Data collection occurred between April and October 2012. Patients were eligible for the study if they received in- or outpatient treatment in one of the three wards during the project period, met the criteria for an F2 diagnosis according to ICD 10 (F20.0 – F29), remained in treatment for at least one week, and expressed explicit interest in attending jogging sessions. This last prerequisite was derived from the theoretical notion and empirical finding that the MCII approach is only expected to support the translation of an individual’s intention into action if he or she is at least moderately committed to the intention (e.g., [40]). Participation in the study was voluntary. Exclusion criteria were severe psychotic symptoms and any medical contraindications for exercising (e.g., cardiovascular or acute infectious diseases).

**Fig. 1:**
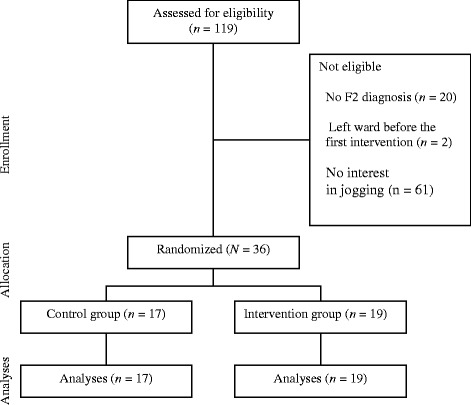
Flow chart of participants’ progress through each stage of the randomized controlled trial

A total of 119 patients were treated at the three wards during the study period, of which 83 patients were not eligible for the study as they had no F2 diagnosis (*n* = 20), left the ward before the first intervention (*n* = 2), or expressed no interest in jogging (*n* = 61). The resulting participation rate was 30.25 %. None of the patients were disqualified for the study because of medical reasons or because they were too psychotic. Figure 1 shows the flow chart of participants.

### Procedure and randomization

During the project period, patients who were admitted to the three wards were screened for inclusion and exclusion criteria. Therapists asked patients in individual therapy sessions whether they wanted to participate in the jogging program and the study. Patients who were explicitly interested and gave their consent were randomly assigned to one of two treatment conditions (MCII or control). The eight trained therapists involved in the study screened potential participants, assigned the treatment condition to patients, and delivered the interventions. To assure proper randomization, the therapists were provided with closed and identical envelopes that contained materials for either the experimental or control condition and were asked to pick one envelope for each patient. These therapists were the only staff with knowledge of the treatment condition, and they were instructed not to reveal the condition. The individuals conducting the exercise sessions, the nursing staff, and the researchers did not know the treatment condition. Prior to the study, the therapists had received training on how to carry out the interventions, the assessments, and the randomization procedure. The therapists implemented the interventions in the participants’ individual therapy sessions. The duration of an individual patient’s study participation depended on the duration of his or her stay in the hospital; after inclusion, patients remained in the study until they were discharged from the clinic. The structure of the exercise program was flexible.

#### Exercise program

We chose jogging as a target behavior because it does not require specific skills or equipment and thus allows patients to continue exercising after their hospital stay. Moreover, jogging was feasible for most participants, and it had been the most widely accepted exercise program among the patients in the respective wards in the past. The sessions were scheduled for 30 minutes. Each session started with joint warm-up exercises. Subsequently, all patients ran a circuit around the psychiatric hospital (approximate length was 1,000 m). Participants were informed that the jogging sessions were not about performance. They were encouraged to run at their own pace and only for as long as they wanted; they could rest or stop at any time and walk back to the ward. At the end of each jogging session, joint cool-down exercises were offered. All sessions were accompanied and monitored by research or nursing staff. As participants might have refused to wear an electronic accelerometer device because of residual symptoms of psychosis, the staff monitored the training in terms of duration of participation (in minutes) and intensity (through participant self-reports). According to these reports, most participants exercised at a moderate level of intensity*.* In each ward, two jogging sessions conducted by research or nursing staff were scheduled every week. We ensured that no conflicting therapies or other events were scheduled for the same time (jogging sessions took place after standard treatment sessions were over for the day).

Patients in the highly structured wards were reminded by the staff immediately before each session, but patients in the autonomy-focused wards received no such reminders. Thus, attending the jogging sessions made higher demands on patients’ self-regulation in the autonomy-focused wards relative to the highly structured wards. Experimental and control participants exercised in the same groups, and the groups were open to patients who were not participating in the study.

### Interventions

#### Goal intention control condition (control)

In the control group, therapists and participants together read an informative text about physical activity. The text emphasized the short- and long-term benefits of exercising and referred to expert opinions. It also contained information about the clinic’s jogging program, including the fact that participants were invited to run at their own pace and could stop or rest whenever they wanted. This information served to point out that regular exercise is a desirable and feasible behavior. In addition, the text highlighted the fact that obstacles may occur that require one to prepare oneself in advance (e.g., motivation problems, tiredness, etc.). After reading the text, participants wrote down three times the goal to attend the jogging sessions. In weeks 2 and 3, participants again wrote down the goal intention twice in order to reinforce the intention.

#### Experimental condition (MCII)

In the experimental group, therapists and participants read the same informational text. In addition, they worked through the MCII strategy. Participants listed three positive outcomes they associated with attending the exercise sessions (e.g., “losing weight”) and three obstacles (e.g., “feeling tired”). After completing the mental contrasting procedure, they identified their most significant obstacle and wrote it down. Together with their therapist, participants then devised a specific solution for this obstacle. Finally, they formulated an if-then plan in the form: “If [obstacle], then I will [solution].” Participants wrote down their if-then plan three times. In weeks 2 and 3, participants again wrote down their if-then plan twice to reinforce the intention. Thus, both groups had equal contact with the therapists with regard to time and attention.

### Measures

#### Primary outcomes

After inclusion in the study, participants’ attendance at scheduled jogging sessions was recorded throughout the remainder of their hospital stay using a set of indicators implemented in previous studies [9]: (a) jogging session attendance (percentage of scheduled sessions attended), and (b) persistence (percentage of weeks in which a participant attended at least one of the two jogging sessions). Because patients were regularly discharged from treatment, we only used the four weeks following inclusion into the study in order to achieve a sufficient sample size. Occasionally, participants had objective and legitimate reasons for missing scheduled jogging sessions, including working probationary, physical injury, appointments with social care workers, visits from family members, and vacation. These sessions were excluded from the evaluation of attendance for these participants.

#### Clinical and control variables

Negative symptoms before inclusion into the intervention program and at discharge from treatment were assessed using the Positive and Negative Syndrome Scale [33]. This instrument is a widely used clinical rating scale for the quantification of symptom severity in psychotic patients. The negative scale includes seven items: blunted affect, emotional withdrawal, poor rapport, passive/apathetic social withdrawal, difficulty in abstract thinking, lack of spontaneity and flow of conversation, and stereotyped thinking. Each item is rated by the clinician on a 7-point Likert scale; the minimum score is thus 7, and the maximum is 49. The instrument has good reliability [32] and internal consistency [34], as well as concurrent and predictive validity [11].

Severity of depression before admission to the study and at discharge from the clinic was assessed with the Beck Depression Inventory (BDI-II [7, 27]). This instrument contains 21 items, with each answer scored on a scale from 0 to 3; higher scores indicate more severe depressive symptoms. The resulting categories are 0–13: minimal depression, 14–19: mild depression, 20–28: moderate depression, and 29–63: severe depression. The instrument has high test-retest reliability and internal consistency, as well as good criterion validity [8, 7].

Physical activity apart from the jogging program was assessed using the short-form version of the International Physical Activity Questionnaire (IPAQ; [15]) before inclusion into the study and at discharge from the clinic. This measure assesses the duration (number of days and hours/minutes per day) that an individual has engaged in walking, moderate, and vigorous activity over the past 7 days. On the basis of these data, Craig and colleagues [15] suggest calculation of a metabolic equivalent (MET)-based IPAQ score by weighting each type of activity by its MET energy requirement: (3.3 x walking duration) + (4 × moderate activity duration) + (8 × vigorous activity duration). In a sample of 35 individuals with schizophrenia, Faulkner et al. [20]) found a reliability coefficient of .68 and a correlation of .37 with an accelerometer, indicating satisfactory validity.

Commitment to attend jogging sessions was assessed with three items: “How likely do you think it is that you will attend the jogging sessions?”, “How important is it to you to attend the jogging sessions?”, and “How disappointed would you be if you failed to attend the jogging sessions?” Participants answered these items using a 7-point Likert scale ranging from 1 (*not at all*) to 7 (*very*). Reliability was good (Cronbach’s Alpha = .82). Participants’ commitment was assessed two times: at study entry and two weeks thereafter (after the MCII or the goal intention control intervention had been implemented).

Participants’ attention and comprehension was rated by the therapists after the intervention session using a three-item scale (“The patient was able to maintain attention throughout the session,” “The patient was able to understand the information,” “Based on your clinical experience, how likely do you think it is that the patient will be able to recall the information from this session tomorrow?”). Answers ranged from 1 (*fully applies*) to 5 (*does not apply at all*). Reliability was high in the present study (Cronbach’s Alpha = .88).

All patients received antipsychotic medication. We assessed the amount of such medication in Chlorpromazine equivalents at the date of inclusion into the study. Each week, therapists asked patients who had missed jogging sessions about the reasons for their absence and documented them. Finally, socio-demographic data were assessed, including age, gender, and education. Measurements of weight at entry and body height also allowed us to compute each participant’s body mass index (BMI).

#### Ethics approval and registration

The study was approved by the ethics committee of the University of Konstanz and registered on ClinicalTrials.gov (registration number: NCT01547026). Patients were only admitted to the study after signing a form to indicate their informed consent.

#### Data analyses

We used SPSS (Version 20) to analyze the data. Parametric tests with Alpha = .05 and effect size measures (i.e., partial eta square) were employed to evaluate group differences. We computed 2 between (Group: MCII vs. control) × 2 between (Setting: autonomy-focused vs. highly structured) ANOVAs as well as Chi^2^ tests (and Fisher’s exact test when preconditions were not fulfilled) to examine the equivalence of the four conditions. To test the Group × Setting hypothesis, we computed 2 between (Group: MCII vs. control) × 2 between (Setting: autonomy-focused vs. highly structured) ANCOVAs in order to adjust the results for differences between conditions in terms of clinical and socio-demographic variables (i.e., BDI, IPAQ, and BMI scores before the intervention as well as age and education), as these variables might have influenced the results. To test our specific hypotheses in greater detail, we computed planned comparisons between the groups for each kind of setting. In order to investigate the change in clinical control variables over time, we computed repeated measurement ANOVAs with pre and post assessments as a within subject factor and intervention condition as a between subject factor. The preconditions for parametric testing were not violated.

## Results

### Equivalence of groups

Of the 97 individuals who fulfilled the first two inclusion criteria (i.e., F2 diagnosis and not leaving the ward before the first intervention), 36 were interested in participating (i.e., a response rate of 37.11 %). The mean age of the actual participants was 30.89 years (*SD* = 11.41). In comparing participants’ ages between conditions, we observed no main effects of group and no Group × Setting interaction effect, both *F*s [1, 33] < 1.83, both *p*s ≥ .186, both partial eta-square (η_p_^2^) < .06, but a main effect of setting, *F* [1, 33] = 20.26, *p* < .001, η_p_^2^ = .39, such that participants in the highly structured setting were older (*M* = 37.20, *SD* = 11.48) than participants in the autonomy-focused setting (*M* = 23.00, *SD* = 4.15). With regard to gender, 11 female and 25 male participants took part in the study. A chi-square analysis revealed no differences in the distribution of the men and women among the four different experimental conditions, *χ*2 (1, *N* = 36) = 6.10, *p* = .107.

### First episode and diagnosis

In relation to the number of patients with first episode disorder and in relation to the type of diagnosis, we found no group and setting main effects and no Group × Setting interaction effect (all *p*s ≥ .294).

### PANSS

With regard to the PANSS scores measured before the intervention, no main effects and no interaction effects were found, all *F*s [1, 33] < 1, all *p*s ≥ .338, η_p_^2^ < .03. The clinicians rated participants in all conditions as having moderate levels of negative symptoms (grand *M* = 19.08*, SD* = 5.97).

### BDI

Concerning the BDI scores assessed before the intervention, we observed no main effects, both *F*s [1, 33] < 2.54, both *p*s ≥ .121, η_p_^2^ < .08, but a Group × Setting interaction effect, *F* [1, 33] = 4.10, *p* = .051, η_p_^2^ = .11. MCII participants reported more symptoms (*M* = 18.00, *SD* = 8.90) than control participants (*M* = 8.13, *SD* = 6.64) in the highly structured setting, *F* [1, 33] = 7.26, *p* = .011, η_p_^2^ = .19, but no such difference between MCII (*M* = 12.71, *SD* = 9.84) and control participants (*M* = 13.89, *SD* = 6.13) was found in the autonomy-focused setting, *F* [1, 33] < 1, *p* = .773, η_p_^2^ < .01.

### IPAQ

With regard to the IPAQ scores assessed before the intervention, we found no main effect of group and no Group × Setting interaction effect, both *F*s [1, 33] < 2.79, both *p*s ≥ .105, η_p_^2^ < .09, but a main effect of setting, *F* [1, 33] = 17.46, *p* < .001, η_p_^2^ = .35, such that participants in the highly structured setting (*M* = 2225.83, *SD* = 1543.76) reported engaging in more physical activity in the week before the intervention than participants in the autonomy-focused setting (*M* = 745.91, *SD* = 490.72).

### Commitment

Concerning participants’ commitment after the intervention, no main effects and no Group × Setting interaction effects were found, all *F*s [1, 33] < 1.57, all *p*s ≥ .220, η_p_^2^ < .05. Participants in all conditions were highly committed to attending the training sessions (grand *M* = 4.92, *SD* = 1.40).

### Attention

Concerning participants’ attention during the intervention sessions, no main effects and no Group × Setting interaction effects were found, all *F*s [1, 33] < 1.57, all *p*s ≥ .220, η_p_^2^ < .05. Clinicians rated participants’ attention during the intervention session and comprehension of the intervention as high in all four conditions (grand *M* = 1.61, *SD* = 0.56).

### Education

With regard to participants’ education, we observed no main effect of group and no Group x Setting interaction effect, both *F*s [1, 33] < 1.97, both *p*s ≥ .171, η_p_^2^ < .06, but a main effect of setting, such that participants in the highly structured setting tended to report more years of education (*M* = 12.75, *SD* = 3.63) than participants in the autonomy-focused setting (*M* = 10.56, *SD* = 1.93), *F* [1, 33] = 3.67, *p* = .064, η_p_^2^ = .10.

### BMI

Finally, concerning Body Mass Index (BMI), we found no main effect of setting and no Group × Setting interaction effect, both *F*s [1, 29] < 1.89, both *p*s ≥ .181, η_p_^2^ < .07, but a main effect of group, such that participants’ BMI before the intervention was higher in the MCII group (*M* = 25.49, *SD* = 3.09) than in the control group (*M* = 23.57, *SD* = 3.10), *F* [1, 29] = 4.41, *p* = .045, η_p_^2^ = .14.

In summary, although the four experimental conditions were comparable for most of the background variables, we found differences in some variables. Consequently, we included these variables (i.e., the BDI, IPAQ, and BMI scores before intervention as well as age and education) in our primary analyses in order to adjust for these differences.

### Primary outcomes: attendance and persistence

#### Attendance

**Fig. 2:**
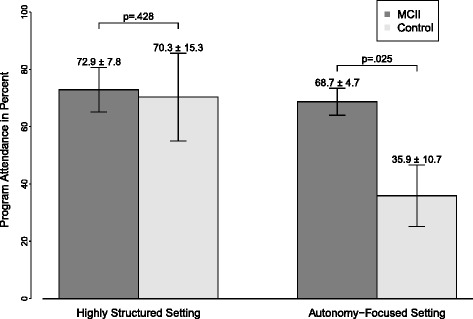
Average program attendance over the course of four weeks in percent by group (MCII vs. control) and setting (autonomy-focused vs. highly structured). Standard errors are represented in the figure by the error bars attached to each column

In order to test our hypothesis that MCII would increase attendance rates relative to the control condition, particularly when applied in an autonomy-focused setting rather than a highly structured setting, we entered the percentage of the total sessions attended into an ANCOVA, adjusting for BDI, IPAQ, and BMI scores before the intervention, as well as for age and education. We observed no main effects of group, which has been our main hypothesis, and setting, both *F*s [1, 24] < 1.61, *p* ≥ .218, η_p_^2^ < .07, but the expected Group × Setting interaction effect, *F* [1, 24] = 5.33, *p* = .030, η_p_^2^ = .19. As expected, in the autonomy-focused setting, MCII participants (*M* = 68.75 %, *SD* = 12.50) attended more sessions than control participants (*M* = 35.94 %, *SD* = 30.21), *F* [1, 24] = 5.72, *p* = .025, η_p_^2^ = .20. In the highly structured setting, however, no differences in the already higher attendance rates were observed between MCII (*M* = 72.92 %, *SD* = 27.09) and control participants (*M* = 70.31 %, *SD* = 43.27), *F* [1, 24] = 0.65, *p* = .428, η_p_^2^ = .03, suggesting a potential ceiling effect. See Table 2 and Fig. 2.

**Table 2:** Number of participants and program attendance for the MCII and control groups by highly structured and autonomy-focused treatment settings

	Week 1	Week 2	Week 3	Week 4	Weeks 1-4
Participants [*n*]					
MCII Group	19	18	16	15	19
Highly structured ward	12	11	11	11	12
Autonomy-focused wards	7	7	5	4	7
Control Group	17	17	16	14	17
Highly structured ward	8	8	8	7	8
Autonomy-focused wards	9	9	8	7	9

Using Fisher’s exact tests, we found no significant differences between groups in terms of the number of patients who remained in the study or left in the first four weeks

#### Persistence

In order to test our analogous hypothesis that MCII increases persistence relative to the control condition, particularly when applied in an autonomy-focused setting rather than a highly structured setting, we entered the percentage of weeks during which the participants attended at least one of the two jogging sessions into an ANCOVA, adjusting for BDI, IPAQ, and BMI scores before the intervention as well as age and education. We observed no main effect of setting, *F* [1, 24] < 1, *p* = .895, η_p_^2^ < .01, but a tendency towards a main effect of group, *F* [1, 24] = 3.44, *p* = .076, η_p_^2^ = .13, such that MCII participants (*M* = 85.94 %, *SD* = 25.77) tended to be more persistent than control participants (*M* = 60.42 %, *SD* = 40.65). This tendency was qualified by a trend towards a Group × Setting interaction effect, *F* [1, 24] = 3.70, *p* = .067, η_p_^2^ = .14. As expected, in the autonomy-focused setting, MCII participants (*M* = 87.50 %, *SD* = 14.43) were more persistent than control participants (*M* = 46.88 %, *SD* = 38.82), *F* [1, 24] = 6.36, *p* = .019, η_p_^2^ = .22. In the highly structured setting, however, no differences in persistence were observed between MCII (*M* = 85.42 %, *SD* = 29.11) and control participants (*M* = 73.96 %, *SD* = 40.20), *F* [1, 24] < 1, *p* = .943, η_p_^2^ < .01.

#### Changes over time in clinical and control variables

Although IPAQ scores tended to decrease over time, we observed no main effects of time for IPAQ or commitment scores, both *F*s < 2.91, *p* > .130. However, we found main effects of time for BDI and PANSS scores. In the total sample, BDI scores significantly dropped over the course of the treatment, from 13.75 (*SD* = 8.52) to 9.77 (*SD* = 9.17), *F* [1, 33] = 8.08, *p* = .008. PANSS scores were also significantly reduced over the course of the treatment, from 19.08 (*SD* = 5.97) to 15.78 (*SD* = 5.34), *F* [1, 33] = 13.79, *p* = .001. Most importantly, no Group × Time interaction effects were found for commitment, PANSS, BDI, or IPAQ, all *F*s < 1, all *p*s > .700.

## Discussion

The present pilot study examined whether the brief CBT self-regulation intervention Mental Contrasting and Implementation Intentions (MCII) could increase physical exercise behavior in a sample of patients with schizophrenia spectrum disorders, particularly in autonomy-focused settings rather than highly structured settings. The findings provide initial support for this assumption. MCII increased attendance rates for scheduled exercise sessions as well as persistence in participation over four weeks in a treatment setting in which patients can and must choose their actions independently, but did not increase the (already higher) attendance rates or persistence in a highly structured treatment setting [39, 43]. Participants in the MCII condition who were not reminded of the exercise sessions (i.e., autonomy-focused wards) participated as much in the sessions as participants who were repeatedly prompted by nursing staff to participate in the sessions (i.e., highly structured ward); that is, constantly reminding patients without further motivational intervention had an effect comparable to that of MCII. This moderation by the type of environmental setting is in line with the theoretical considerations developed by Oettingen and colleagues, who outlined the importance of the social context for successful goal pursuit in early stages [39, 43]. Notably, adherence rates during the study were rather high. Overall, participants attended 61.75 % of the offered exercise sessions. In 73.44 % of the possible weeks, they attended at least one of the two weekly jogging sessions (persistence). These rates resemble those observed in other trials, such as the Diabetes Prevention Program (DPP; [35]), in which 74 % of individuals with impaired glucose tolerance met the goal of at least 150 min of physical activity per week after 24 weeks, and the Look AHEAD trial [37], in which overweight or obese patients with type 2 diabetes attended an average of 84 % of the possible group and individual lifestyle intervention sessions during the first year. Although participants’ commitment ratings did not change over the course of the study, their physical activity apart from the jogging sessions tended to decrease over time in both conditions. However, this decline is compatible with the frequently observed post-psychotic fatigue, persistent negative symptoms, and sedating medication effects [4].

### Implications

The present findings have clear practical implications. Although many psychiatric institutions promote physical activity as an adjunct treatment and it has been argued that physical activity interventions should become a routine component of comprehensive psychiatric care for individuals with mental illness (e.g., [47]), physical activity interventions for this group of patients are typically assumed to require a structured clinical setting and thus entail high costs (e.g., [9]). Moreover, even in relatively controlled settings, prior physical activity interventions found only moderate effects. For example, Archie et al. [6] examined whether free access to a fitness center could increase exercise program adherence, finding that increasing motivation by providing free access to exercise facilities was not sufficient to reduce the intention-behavior gap in individuals with schizophrenia. The present findings suggest that exercise interventions can benefit from adding self-regulatory and planning strategies, especially in outpatient and community settings in which patients live autonomously (e.g., assisted living). The present study justifies more research on self-regulation interventions like MCII in order to develop therapeutic tools that can be easily applied and are cost-efficient in settings without a highly structured environment.

The benefits of the MCII brief intervention were not diminished by any adverse effects on clinical variables such as negative symptoms of schizophrenia or symptoms of depression. All patients received standard treatment for psychiatric symptoms, and all improved during the project period; MCII had no additional effect on their psychiatric symptoms. However, given the short treatment period, the main focus of the present study was on attendance and persistence in the jogging program rather than on examining the effect of exercise behavior on psychiatric symptoms. In addition, our randomization strategy served to level out differences in participants’ symptoms at baseline between the conditions, as such differences might also influence participation in exercise sessions. Although we did not find differences for most of the symptoms, we observed differences in the depression symptoms. However, we adjusted for the differences observed between the groups at baseline by including them as covariates in our analyses. Most importantly, the beneficial effects of MCII in the autonomy-focused ward were evident whether we included the covariates or not.

With regard to research on self-regulation interventions, the present study implies that MCII can be effectively applied in clinical populations with deficits in cognition, perception, affect, and volition. In fact, the severity of symptoms did not moderate the approach’s beneficial effects on physical exercise in the present study. These findings are also in line with previous research. For example, Brandstätter et al. [12] found support for the effectiveness of the implementation intention strategy in improving goal attainment in schizophrenia patients in a laboratory-based reaction time study. These findings complement the present study, as they show the effectiveness of the implementation intention self-regulation strategy not only in a real-life treatment program but also in a controlled laboratory setting with a fine-grained response time measure. In addition, the present study applies theory-based research on motivational processes to the commencement and continuation of physical activity in patients with schizophrenia, a research gap that has been highlighted by Vancampfort et al. [59]. Thereby, our MCII brief intervention complements and extends other motivational intervention approaches addressing exercise in patients with schizophrenia that have been derived from Self-Determination Theory [17] and from Goal Setting Theory [36]. Self-Determination Theory focuses on the degree to which a behavior is self-motivated and self-determined. In line with the assumptions of this theory, patients’ reported regulation has been found to be correlated with their physical activity: Whereas autonomous regulation was positively correlated with physical activity, external regulation and amotivation were negatively correlated with physical activity [59]. Moreover, negative symptoms have been found to be associated with lower autonomous motivation to engage in physical activity [62]. In a recent review of qualitative articles, Soundy et al. [56] highlighted the significance of this theoretical approach and especially of physical activity programs, as they may help individuals to regain autonomy in other parts of their lives – for example, by increasing social competence and self-confidence. Goal Setting Theory focuses on the phrasing of goals. In line with the assumptions of this theory, setting specific rather than broad goals was found to promote exercise program attendance in schizophrenic patients [64]. Thus, in addition to the importance of the source of the motivation to exercise and the formulation of the exercising goals, the self-regulation of individuals’ exercise goal setting via mental contrasting and of their goal striving via the formation of implementation intentions plays an important role when it comes to establishing strong goal commitment and to effectively translating goal intentions into actual behavior. Although the specific prerequisites for the successful application of self-regulation interventions such as the MCII strategy warrants further research, the present study is a first step towards identifying the approach’s applicability to clinical populations. Assuming that these findings will be replicated and extended, future research could examine whether the MCII self-regulation strategy could also be used to promote other illness-related behaviors for people with schizophrenia (such as such as consistently taking their medication, attending therapy sessions, and not using drugs or drinking alcohol) or for other client groups.

### Limitations

The limitations of this study include the mix of experimental and quasi-experimental manipulations of independent variables. The degree of patient autonomy (i.e., the intensity with which the staff asked and reminded patients to participate in the jogging sessions) was a quasi-experimental variation. However, the fact that a pilot study has shown initial evidence of the moderating effect of the degree of autonomy in the living environment justifies the experimental varying of this variable in future studies (e.g., as an additional control condition). Other limitations of this study include the small sample size and the relatively broad diagnostic group. Moreover, the current study did not manage to achieve perfect randomization: Patients in the intervention group tended to be older and had higher BMIs, more severe depression, and more extensive formal education. Although we statistically adjusted for these differences, comparability between our patient groups may not have been fully achieved. Future research might replicate the present study using a larger sample of individuals with schizophrenia; in addition, researchers could implement a more detailed assessment of psychiatric symptoms including positive and manic symptoms as well as anxiety, and could utilize objective-based measures to improve the quality of the measurement of physical activity apart from the jogging program (e.g., [55]). However, the double-blinded randomized and controlled design and the robustness of the results strengthen the confidence in the observed effects. Another limitation is the fact that we assessed prescribed medications only at the baseline. Although it is possible that participants changed their medications during the study, entailing substantial effects on affect, volition, and cognition (for a meta-analysis, see [50]) as well as potentially inducing several undesired side-effects (e.g., [5]), any changes in medication should be distributed equally between the two experimental groups, such that potential differences should not be able to explain the improved attendance and persistence of the MCII group relative to the goal setting control group. As another limitation, experimental and control participants exercised in the same group. Contamination of treatment conditions was thus theoretically possible (e.g., by patients motivating each other to participate – for example, by making plans amongst themselves to participate jointly in the jogging sessions and reminding each other). However, as such influence should have equal treatment effects, it cannot explain the observed differences between the control and experimental conditions. In addition to forming separate exercise groups, future research might seek to assess the intensity of participants’ exercise using a more fine-grained measure than the subjective ratings that were applied in the present study. We also limited our exercise program to a single type of exercise, namely jogging. This could affect the generalizability of our data, in the sense that patients with a preference for jogging are a specific group of patients. However, jogging had been the most widely accepted form of physical activity at the clinics prior to our study, and the threshold for participation is low. We also believe that the underlying problem – patients’ intention-behavior gap – is the same for any kind of exercise behavior. Thus, we believe that our data on MCII, which shows that the strategy helped to bridge this gap, can be generalized to a broader range of exercise behaviors. Nevertheless, future studies should include different types of exercise as target variables. Finally, we only included patients who expressed their wish to participate in the exercise program. It would be desirable to include all patients and to motivate even those who do not express a wish to participate in physical activity. Despite these limitations, the present study demonstrates that MCII applied as a brief intervention can be especially effective in situations in which patients are not closely guided (Additional file 1).

## Conclusions

The weight gain produced by antipsychotic drugs [5], increased sleeping time, and more sedentary activities [28, 48, 61] are important contributors to the higher risk of medical illness in individuals with schizophrenia. As exercising has been found to effectively combat these health risks (e.g., [25, 51]), interventions to promote exercising in this population are highly necessary. This study found first evidence that MCII intervention is a promising way to successfully promote physical activity among people with schizophrenia spectrum disorders, justifying further research. We also found evidence of a moderating effect related to the type of setting in which patients live: Motivational strategies designed to address the intention-behavior gap work differently in living environments with different degrees of autonomy. This theoretically well-supported observation calls for systematic empirical research. The present study contributes to the research on new directions for promoting behavior-change processes in individuals with severe mental illness. Self-regulation strategies, specifically the combination of strategies targeting goal setting as well as goal striving, might constitute a set of time- and cost-efficient tools that can simultaneously benefit the health of patients and tight public budgets.

## Additional file

Additional file 1:
**CONSORT 2010 checklist of information to include when reporting a randomised trial*.**
